# Need for Timely Paediatric HIV Treatment within Primary Health Care in Rural South Africa

**DOI:** 10.1371/journal.pone.0007101

**Published:** 2009-09-22

**Authors:** Graham S. Cooke, Kirsty E. Little, Ruth M. Bland, Hilary Thulare, Marie-Louise Newell

**Affiliations:** 1 Africa Centre for Health and Population Studies, University of KwaZulu-Natal, KwaZulu-Natal, South Africa; 2 Imperial College London, London, United Kingdom; 3 University College London Institute of Child Health, London, United Kingdom; 4 Division of Developmental Medicine, University of Glasgow Medical Faculty, Glasgow, United Kingdom; University of Stellenbosch, South Africa

## Abstract

**Background:**

In areas where adult HIV prevalence has reached hyperendemic levels, many infants remain at risk of acquiring HIV infection. Timely access to care and treatment for HIV-infected infants and young children remains an important challenge. We explore the extent to which public sector roll-out has met the estimated need for paediatric treatment in a rural South African setting.

**Methods:**

Local facility and population-based data were used to compare the number of HIV infected children accessing HAART before 2008, with estimates of those in need of treatment from a deterministic modeling approach. The impact of programmatic improvements on estimated numbers of children in need of treatment was assessed in sensitivity analyses.

**Findings:**

In the primary health care programme of HIV treatment 346 children <16 years of age initiated HAART by 2008; 245(70.8%) were aged 10 years or younger, and only 2(<1%) under one year of age. Deterministic modeling predicted 2,561 HIV infected children aged 10 or younger to be alive within the area, of whom at least 521(20.3%) would have required immediate treatment. Were extended PMTCT uptake to reach 100% coverage, the annual number of infected infants could be reduced by 49.2%.

**Conclusion:**

Despite progress in delivering decentralized HIV services to a rural sub-district in South Africa, substantial unmet need for treatment remains. In a local setting, very few children were initiated on treatment under 1 year of age and steps have now been taken to successfully improve early diagnosis and referral of infected infants.

## Introduction

Within the broader global HIV epidemic, paediatric HIV infection remains a major cause for concern. UNAIDS estimates that although only accounting for approximately 6% of all HIV infected individuals, children under 16 years of age account for 12% of new infections and 13% of HIV related deaths [1]. The relatively high proportion of deaths illustrates the need for timely access to quality care and treatment [2], a need emphasized by recent data on the benefits of early treatment in infants [3].

Nowhere are the challenges of preventing and treating paediatric infection more acute than in South Africa, home to more than one in seven of the world's HIV infected population [1], and where there is no evidence to date of substantial reductions in the prevalence of HIV in antenatal clinics [4]. Mother-to-child transmission (MTCT) accounts for the large majority of paediatric infections and PMTCT targets the peri-partum period. We have previously estimated the number of children likely to be eligible for treatment in South Africa up to end of 2003 using a novel model [5]; of the 758,595 children estimated to have acquired HIV infection between 1993–2003, approximately half were predicted to be alive and ultimately in need of HAART at the time of widespread treatment roll-out.

Although early treatment has been shown to reduce mortality in vertically-infected infants [3], identification and early referral of infected infants in the first weeks or months of life remains a challenge in most settings. With the advent of antiretroviral roll-out, HIV programme managers at a local level increasingly need to make difficult decisions on how to allocate scarce resources to best meet the needs of the population they serve [2]. Here we apply the existing model to a well characterized rural sub-district of KwaZulu-Natal using locally derived data to assess the number of children expected to be alive and eligible for treatment on the basis of clinically advanced disease and use the estimates to assess the performance of a decentralized paediatric HIV service.

## Methods

Hlabisa sub-district lies in northern KwaZulu-Natal within the Umkhanyakude district which is one of the most deprived districts within South Africa [6]. The area is largely rural. The sub-district hosts a demographic surveillance site (DSS) in which detailed demographic, economic and health data on the local population have been collected since 2000; the surveillance covers about half of the population of the sub-district [7].

The Department of Health HIV Treatment and Care programme in the Hlabisa sub-district first began treating patients in late 2004. The programme is run in partnership with the Africa Centre for Health and Population Studies, University of KwaZulu-Natal (www.africacentre.com). This analysis addresses the period from programme inception until 2008. Whilst initially based in the district hospital, the service is now decentralised to a community healthcare centre (CHC) and all primary health care centres (PHCs) within the wider Hlabisa sub-district [8]. Paediatric care in the local service was integrated with adult services at all sites and care was provided by trained healthcare workers, primarily counsellors and nurses with regular medical support. Treatment was provided within South African government guidelines which use a combination of clinical and CD4 criteria for initiation of treatment [9].

PMTCT services in the area started in August 2001 at one clinic and by August 2002 were rolled out to all primary health care clinics in the sub-district[10]. Single dose nevirapine was provided to mothers and infants, and infant feeding counselling provided. After 2004, when the HIV treatment programme was established, women with CD4 counts <200 cells per ml were referred for treatment for their own health. South African PMTCT guidelines were revised in late 2007 to include zidovudine for HIV infected pregnant women from 28 weeks gestation in line with WHO recommendations [11] and implemented in April 2008.

Within the partnership, the Africa Centre is the custodian of a database relating to patients enrolled in the HIV treatment programme; the number of infected children in care within the programme were provided through the routine monitoring and evaluation process. Patients gave written informed consent on enrollment. Ethics approval for use of programmatic data was received from the Department of Health, Pietermaritzburg and Biomedical Research Ethics Committee of the University of KwaZulu-Natal.

A deterministic mathematical modeling approach was employed to estimate the number of HIV infected children who should be accessing HIV care and treatment within the sub-district. The design of the model and the assumptions contained within it has been described in detail previously [5]. Variables required for the model were: population size, antenatal HIV prevalence, birth rate, uptake of PMTCT services by pregnant women, proportion of mothers breastfeeding during post partum period, median duration of breast feeding, survival with and without HAART and the proportion of HIV positive children taking up treatment. Data used for the model are presented in Appendix S1; where available, local data for the time period were used. The population of the sub-district was estimated from census data at 212,000 and assumed to be constant throughout the period in question. Birth rates for the period 2000 to 2007 were computed using data from the Africa Centre Surveillance from the registered annual births divided by the annual person years of exposure. The number of births for 1999 and 1998 (before the start of the Africa Centre surveillance) were obtained by reverse projecting the population of children who were 1–2 year and 2–3 year old in 2000, respectively. To obtain the mid-year population, the total population for 2000 was also reverse projected to 1999 and 1998. Birth rates from the demographic surveillance population were assumed to be representative of the wider sub-district. The median duration of breastfeeding was assumed to be constant throughout the study period at five months. Antenatal prevalence data were taken from Department of Health estimates for Hlabisa sub-district [4] as was uptake of single dose nevirapine (sdNVP) by HIV positive mothers [6] and extrapolated forward assuming constant coverage between 2004–7, consistent with local data. Data for breastfeeding were estimated from a large local study of vertical transmission [12], [13]. HAART eligibility was based on clinical disease progression [14] and the estimated number of children with moderate and severe clinical disease (equivalent to WHO stage 3 or 4) in a particular age group [5]. In the first year of life the total number of infants (ie those aged under 1 year) with advanced clinical disease equates to the estimated number progressing to severe disease; in later years the estimated total number is a cumulation of those surviving from previous years plus those developing severe disease in a given year, after applying the age-category specific mortality rate [5]. As the mortality rate in the first year of life is very high, and disease progression very rapid, the disease progression and mortality rates are applied on a monthly basis. Without rapid diagnostics, or treatment on the basis of presumptive diagnosis, the majority of children who progress to serious disease in their first year will not be diagnosed and not access effective treatment before they die – consequently, very small numbers of infants are estimated to be requiring treatment. This effect remains throughout the cohort, although becomes less pronounced with age.

Sensitivity analyses addressed varying assumptions relating to PMTCT uptake and regimen, and timely diagnosis of infants born to HIV infected mothers, with appropriate and rapid referral into the treatment programme. We assume that the rate of PMTCT uptake remains constant with 54% of women receiving sdNVP (overall MTCT rate  = 17.0%), we then examined the impact of adding Zidovudine to the sdNVP, both at 54% and 100% coverage (overall MTCT rates or 14.9% and 8.8% respectively). We also explored the potential effect if women with a CD4 count of 350 or less would be eligible for HAART by assuming a 50% coverage of HAART initiated during pregnancy, with the remaining women with a CD4 count above 350 cells receiving sdNVP (overall MTCT  = 7.9%). Breastfeeding rates remained unaltered for all the sensitivity analyses, although HAART is assumed to lower the post-natal transmission rate.

## Results

The estimated number of HIV exposed and infected children born annually increased between 1997 and 2000 (Table 1), but subsequently declined following a reduction in the birthrate [15], the introduction of PMTCT services, and changes in infant feeding practices which would have reduced the risk of MTCT.

**Table 1:** Estimated annual number of infants born to HIV infected women (exposed) and numbers of infected and uninfected infants.

Year	Exposed infants	Infants infected	Infants uninfected
2007	1837	313	1,524
2006	2249	386	1,864
2005	2642	456	2,186
2004	2588	449	2,139
2003	2256	439	1,817
2002	2321	491	1,830
2001	2523	567	1,957
2000	2843	653	2,191
1999	2519	578	1,940
1998	2490	572	1,918
1997	2142	492	1,650

The estimated total number of surviving HIV-infected children up to 10 years of age in 2007 was 2,561 (Table 2), of whom 521 would be in need of immediate antiretroviral treatment (ART) under the model assumptions which relate to the treatment guidelines during the period of study. Changing the model assumption to all infants being diagnosed promptly (and thus some receiving treatment earlier), the total number of children estimated to be in need of treatment would rise to 576.

**Table 2:** Estimated number of surviving HIV infected children requiring treatment by age, 2007 [5].

AGE (years)	Total number estimated to be alive	Estimated number Alive and MSD^1^ free	Estimated number requiring Treatment
1	334	285	49
2	388	300	88
3	315	245	70
4	250	207	42
5	239	202	37
6	242	198	44
7	258	200	57
8	207	158	49
9	186	140	46
10	144	106	38
	2,561	2,040	521

^1^MSD  =  Infected children with moderate/severe disease who would meet treatment eligibility criteria.

The number of infected children who would be estimated to be alive declines slowly with age, with 334 predicted to survive to their first birthday and 144 reaching their 10^th^ birthday in 2007 (Table 2). The number of children predicted to be requiring antiretroviral treatment varies by age: 207/1037 (19.9%) of surviving HIV infected children aged 3 years or younger were predicted to require treatment depending on whether an immediate diagnosis of HIV was made or not; 115/731 (15.7%) of surviving children aged 4–6 years and 190/795 (23.8%) of surviving children 7–10 years of age. These variations are a reflection of the local dynamics of the epidemic.

To address the issue of early diagnosis and treatment of vertically-infected infants, we estimated the number of HIV infected infants born each year, which shows a steady annual decline (Table 1). If the uptake of the 2007 PMTCT regimen, sdNVP, remained constant along with all variables, through the following year, we would predict that 299 infected children would be born to 1755 HIV positive mothers during 2008. If we further allow for the addition of Zidovudine to the PMTCT regimen, as per 2008 SA guidelines, the number of infected children would be reduced to 262. Results from further sensitivity analyses show that should it be possible to increase uptake of Zidovudine/sdNVP to 100%, an estimated 152 children would be infected – a reduction of 49.2% from the 299 infected children predicted. Assuming that pregnant women with a CD4 count of 350 cells per ml or less would be eligible for HAART, with an associated reduction in the rate of MTCT, would in 2008 reduce the number of infected children to 138, a 62% reduction. These figures assume all other variables to be constant.

From programme inception until the end of 2007, a total of 346 children under the age of 16 years were recorded as initiating ART, 184/346 (53.1%) were male. The number of children initiating treatment for the periods 2004/2005, 2006 and 2007 were 59, 103 and 184 respectively. A total of 245/346 initiated treatment aged 10 or younger. Compared to the lowest estimated figure of 521 needing treatment (assuming conservative initiation criteria in place during the period of study), these figures suggest two thirds of infants requiring HAART had initiated treatment. Inclusion of infants less than one was rare, with only one in 2006 and one in 2007. However, in 2008 with the establishment of a family clinic and improvements in the service, there were 41 infants less than one year of age initiated on treatment and these results will be presented more fully in future work.

## Discussion

Antiretroviral roll-out is maturing in South Africa and by the end of 2007 national figures from South Africa estimated that 32,060 children under the age of 15 had been initiated on treatment within the public sector [16]. One of the many challenges faced by the health system is the high number of children in need of antiretroviral treatment in areas of high HIV prevalence and the demands in reaching all HIV positive mothers with a package of care to prevent mother-to-child transmission. The failure to meet the demand for care and treatment is part of the reason that there has been little progress in achieving one of the key Millenium Development Goals in sub-Saharan Africa, namely reducing the mortality rate for children under five by two-thirds by 2015 [17].

The ongoing scale of the epidemic poses enormous challenges for programme managers trying to meet the need of their populations with limited resources and for whom it is important to have some estimate of the numbers of individuals who should be accessing care within local settings [2]. Here we apply a model that has been described previously [5] to a local population and find that by the end of 2007 approximately two thirds of the number of children predicted to need treatment had started HAART.

The numbers produced by this method of estimation should only be considered an approximation of the reality on the ground. The extent to which uptake of treatment in the local population as a whole can be reliably established from the data shown here is limited by a number of factors. The public sector service, whilst the dominant provider in many settings, including the one described here, is not alone in providing treatment; GPs, NGOs, private practitioners/paediatricians and employers (for parents) amongst others also contribute to meet the needs of the population. As such, the figures for treatment uptake here are likely to represent a lower estimate of the actual number of individuals initiated on treatment in the local area, although the evidence available suggests the use of private healthcare services in the area is low [8].

In addition, the estimates of need in this population should be considered conservative, with approximately 20% of infected individuals predicted to be in need of treatment in this setting. These estimates were based on guidelines in place at the time and reflect need as defined by guidelines. Recent data emerging from the CHER study on the benefits of early treatment [3] and subsequent changes to South African paediatric treatment guidelines [18], emphasize the well-recognised need to improve timely access of children to care and treatment, particularly through improved systems of early diagnosis [19], [20]. What can be seen from the local data is that compared to the proportion of children estimated to be in need of treatment within each age category, the number of children initiating treatment is broadly similar across age ranges, except in those aged under one year where there is a more dramatic short-fall. It is of interest to note that through targeted efforts within the programme in the identification and rapid referral of infected infants, the numbers of infants under one year of age in the programme has dramatically increased during the latter half of 2008. Initiatives included a one-day workshop for all HIV counsellors on paediatric eligibility for ART and the importance of rapid referral of HIV-infected infants; training to increase awareness amongst nurses of the clinical symptoms and signs associated with HIV in infancy; testing of all children admitted to hospital where consent given; using opportunities presented during immunization and vitamin A campaigns at primary health care facilities to check health cards of all children to ensure their HIV status known. This shows that substantial improvements can be made relatively rapidly, where data and resources are available to inform intervention. However, modelling of future treatment will need to have greater focus on those under one year of age, particularly with new WHO recommendations that all children under 1 year of age should receive ART irrespective of their clinical or immunological status [21].

Whether the figures for uptake within the local programme here are indicative of “good” or “bad” performance is not a straightforward, nor perhaps a very helpful question [22]. There are very little data for comparison with other programmes in different healthcare settings, though this methodology could be applied elsewhere as one form of comparison. One could argue that in a rural environment with limited healthcare resources where care is largely delivered through a decentralized system of primary healthcare clinics, that to achieve the levels of uptake seen here is important progress. However, whilst currently manageable, even these predicted numbers of HIV infected children require substantial resources [2]. Even using our most conservative estimate here of 152 infants born with HIV each year, if all were diagnosed and treated in a timely manner, resources will soon be stretched if there is no further change in adult HIV prevalence and fertility rates.

As with all healthcare initiatives in resource poor settings, attention has to be given to sustainability, quality of care and the opportunity costs of prioritizing one area of care. The development of paediatric care and treatment services will need to be explicitly linked into broader prevention efforts including interventions targeting fertility and aimed at keeping HIV negative individuals, particularly adolescents, negative. How this can best be done needs to be a research priority. One such approach is to identify ways in which the provision of family services, can provide an avenue into the broader challenge of adult HIV transmission in the wider population. Expanding HIV testing and encouraging all to know their status are cornerstones of prevention efforts and, engagement with paediatric services offers one route to encourage other family members, particularly fathers, who might not be aware of their HIV status to test, and if necessary enter care.

The data presented here show that despite expansion of services and efforts towards early diagnosis and care for infected children, there is still a significant unmet need for treatment in this setting, even when services are available at primary care facilities. It is possible through targeted efforts to rapidly improve access, however this will continue to challenge limited healthcare resources for the foreseeable future, particularly with greater emphasis on early diagnosis and treatment in infants, and this should further encourage prevention efforts both in mother to child transmission and in the general population.

## Supporting Information

Appendix S1 Percentage of women attending antenatal clinic who test positive for HIV; estimates taken from South African Dept of Health figures[4]. 2 Number of live births per 1,000 population; estimates from Africa Centre Surveillance data 3 Percentage of HIV infected pregnant women who receive either no treatment or single-dose NVP to prevent MTCT; estimates based on mothers and infants receiving treatment and taken from South African Department of Health Estimates for 2003/4 [6] and extrapolated forward assuming constant coverage 4 Percentage of HIV infected mothers who a, never breastfed their infant, b, breastfed their infant for <6 months, or c, breastfed their infant for more than 6 months; estimates extrapolated from local study of infant feeding behaviours [12], [13] 5 Percentage of HIV infected children who have access to a, no treatment, or b, PCR testing, co-trimoxazole prophylaxis and ART when required; estimates from Africa Cente Surveillance data
(0.04 MB DOC)
