# Correction: Need for Timely Paediatric HIV Treatment within Primary Health Care in Rural South Africa

**DOI:** 10.1371/annotation/4134e87b-0199-4457-8b3d-56ddf4f475e4

**Published:** 2009-09-24

**Authors:** Graham S. Cooke, Kirsty E. Little, Ruth M. Bland, Hilary Thulare, Marie-Louise Newell

The last sentence of the second paragraph in the Discussion section was incorrect. The correct sentence is: "Here we apply a model that has been described previously [5] to a local population and find that by the end of 2007 approximately half of the number of children predicted to need treatment had started HAART.

