# Correction: Need for Timely Paediatric HIV Treatment within Primary Health Care in Rural South Africa

**DOI:** 10.1371/annotation/dbee123b-8386-4f30-b4a7-0eb4bb9f7464

**Published:** 2009-09-24

**Authors:** Graham S. Cooke, Kirsty E. Little, Ruth M. Bland, Hilary Thulare, Marie-Louise Newell

The fourth sentence of the last paragraph of the Results section was incorrect. The correct sentence is: Compared to the lowest estimated figure of 521 needing treatment (assuming conservative initiation criteria in place during the period of study), these figures suggest approximately half of infants requiring HAART had initiated treatment.

