# Supplementary material for: Need for Timely Paediatric HIV Treatment within Primary Health Care in Rural South Africa
Source: PLoS One. 2009 Sep 22;4(9):e7101. doi: 10.1371/journal.pone.0007101 (PMC2742735; doi:10.1371/journal.pone.0007101)
Supplement: Appendix S1 — Percentage of women attending antenatal clinic who test positive for HIV; estimates taken from South African Dept of Health figures[4]. 2 Number of live births per 1,000 population; estimates from Africa Centre Surveillance data 3 Percentage of HIV infected pregnant women who receive either no treatment or single-dose NVP to prevent MTCT; estimates based on mothers and infants receiving treatment and taken from South African Department of Health Estimates for 2003/4 [6] and extrapolated forward assuming constant coverage 4 Percentage of HIV infected mothers who a, never breastfed their infant, b, breastfed their infant for <6 months, or c, breastfed their infant for more than 6 months; estimates extrapolated from local study of infant feeding behaviours [12], [13] 5 Percentage of HIV infected children who have access to a, no treatment, or b, PCR testing, co-trimoxazole prophylaxis and ART when required; estimates from Africa Cente Surveillance data (0.04 MB DOC) [file pone.0007101.s001.doc]

| Year: | 2007 | 2006 | 2005 | 2004 | 2003 | 2002 | 2001 | 2000 | 1999 | 1998 | 1997 |
| --- | --- | --- | --- | --- | --- | --- | --- | --- | --- | --- | --- |
| Antenatal HIV Prevalence ^1^ | 36 | 38 | 39 | 41 | 38 | 37 | 34 | 36 | 33 | 33 | 27 |
| Birth rate (per 1000)^2^ | 24.1 | 28.3 | 31.9 | 30.0 | 28.4 | 30.0 | 35.0 | 37.2 | 36.5 | 36.1 | 37.4 |
| Percentage of Women on^3^: |  |  |  |  |  |  |  |  |  |  |  |
| No treatment | 46.0 | 46.0 | 46.0 | 46.0 | 66.8 | 83.4 | 95.8 | 100.0 | 100.0 | 100.0 | 100.0 |
| Single-dose NVP only | 54 | 54 | 54 | 54 | 33 | 17 | 4 | 0 | 0 | 0 | 0 |
| Total % | 100.0 | 100.0 | 100.0 | 100.0 | 100.0 | 100.0 | 100.0 | 100.0 | 100.0 | 100.0 | 100.0 |
| Percentage of infected women breastfeeding for:^4^ | |  |  |  |  |  |  |  |  |  |  |
| 0 months | 26.0 | 22.3 | 18.6 | 14.9 | 11.1 | 7.4 | 3.7 | 0.0 | 0.0 | 0.0 | 0.0 |
| < 6 months | 40.0 | 34.3 | 28.6 | 22.9 | 17.1 | 11.4 | 5.7 | 0.0 | 0.0 | 0.0 | 0.0 |
| > 6 months | 34.0 | 43.4 | 52.9 | 62.3 | 71.7 | 81.1 | 90.6 | 100.0 | 100.0 | 100.0 | 100.0 |
| Total % | 100.0 | 100.0 | 100.0 | 100.0 | 100.0 | 100.0 | 100.0 | 100.0 | 100.0 | 100.0 | 100.0 |
| Paediatric treatment available^5^ |  |  |  |  |  |  |  |  |  |  |  |
| No treatment | 0.0 | 0.0 | 0.0 | 46.0 | 66.8 | 83.9 | 95.8 | 100.0 | 100.0 | 100.0 | 100.0 |
| Early intervention Co-trim and ART | 100 | 100 | 100 | 8 | 0 | 0 | 0 | 0 | 0 | 0 | 0 |
| Total % | 100.0 | 100.0 | 100.0 | 100.0 | 100.0 | 100.0 | 100.0 | 100.0 | 100.0 | 100.0 | 100.0 |
